# Global Status of Bufavirus, Cosavirus, and Saffold Virus in Gastroenteritis: A Systematic Review and Meta-Analysis

**DOI:** 10.3389/fmed.2021.775698

**Published:** 2022-01-13

**Authors:** Mohammad Hossein Razizadeh, Alireza Khatami, Mohammad Zarei

**Affiliations:** ^1^Faculty of Medicine, Department of Virology, Iran University of Medical Sciences, Tehran, Iran; ^2^Renal Division, Brigham and Women's Hospital, Harvard Medical School, Boston, MA, United States; ^3^John B. Little Center for Radiation Sciences, Harvard T.H. Chan School of Public Health, Boston, MA, United States

**Keywords:** Bufavirus, Saffold virus, Cosavirus, gastroenteritis, meta-analysis

## Abstract

**Background:** Bufavirus (BuV), Human Cosavirus (HCoSV), and Saffold (SAFV) virus are three newly discovered viruses and have been suggested as possible causes of gastroenteritis (GE) in some studies. The aim of the present study was to estimate the overall prevalence of viruses and their association with GE.

**Methods:** A comprehensive systematic search was conducted in Scopus, Web of Science, PubMed, and Google scholar between 2007 and 2021 to find studies on the prevalence of BuV, HCoSV, and SAFV viruses.

**Result:** Meta-analysis of the 46 included studies showed the low prevalence of BuV (1.%, 95% CI 0.6–1.5%), HCoSV (0.8%, 95% CI 0.4–1.5%), and SAFV (1.9%, 95% CI 1.1–3.1%) worldwide. Also, no significant association between these viruses and GE was observed. BuV was isolated from patients with GE in Africa, while SAFV was more common in Europe. BuV1 and BuV2 have the same prevalence between the three identified genotypes of BuV. HCoSV-C was the most prevalent genotype of HCoSV, and SAFV2 was the commonest genotype of SAFV. All of these viruses were more prevalent in children older than 5 years of age.

**Conclusion:** This was the first meta-analysis on the prevalence and association of BuV, HCoSV, and SAFV with GE. While no significant association was found between infection with these viruses and GE, we suggest more studies, especially with case-control design and from different geographical regions in order to enhance our knowledge of these viruses.

## Introduction

Gastroenteritis (GE) is one of the most common illnesses in both children and adults worldwide. The high importance of GE is due to both high morbidity and mortality and also the financial burdens of the disease. Children, the elderly, and immunocompromised individuals are at higher risk of severe GE (1). Infectious agents, particularly viruses are the main cause of GE worldwide (2). Before the implication of Rotavirus vaccination, Rotavirus was the leading cause of viral GE, while other enteric viruses, such as Noroviruses, Astroviruses, and Human adenoviruses, are now the most prevalent viruses causing GE (3). Besides the aforementioned enteric viruses, the list of enteric viruses is continuously growing due to the discovery of emerging viruses (4, 5). Since still 40% of cases of GE are of unknown etiology (6), these newly discovered viruses may likely be involved in causing the GE (7).

The *Parvoviridae* family consists of small, non-enveloped, icosahedral-shaped viruses, which have a single-stranded DNA genome. Members of this family can infect both vertebrates and invertebrates (8). For about 3 decades, Parvovirus B19 was taught to be the only human pathogen in this family (9). In 2005, Human bocavirus 1 was isolated from the nasopharyngeal swab of children with respiratory symptoms. Since 2009, three other types of the virus, named Human bocavirus 2–4, have been isolated from a stool specimen of children with or without GE (10). In 2012, the metagenomic survey of stool samples of children with acute diarrhea in Burkina Faso resulted in the discovery of a new member of this family, which was named Bufavirus (BuV) (9). Human BuVs belong to the genus *Protoparvovirus*, and, so far, three genotypes of Human BuV have been identified (11).

The *Picornaviridae* family contains non-enveloped, icosahedral-shaped viruses with a positive-sense single-stranded RNA genome (12). Unlike the *Parvoviridae*, viruses in the *Picornaviridae* family are not able to infect invertebrates (13). This family contains a growing number of viruses, which cause a variety of diseases that can affect different organs of the body. In 2007, a new member of this family was isolated from a child with a fever of an unknown origin in the United States. This virus was later named Saffold virus (SAFV); this name was derived from the lead author of the research, Morris Saffold Jones. Phylogenetic analysis showed that this virus is closely related to *theilovirus* species in the *Cardiovirus* genus of this family (14). Since then, eight genotypes of SAFV have been identified (15). The other virus in this family is the Cosavirus (CoSV), which was discovered in 2008 in pediatric patients with acute flaccid paralysis and later found in patients with GE (7). These three novel viruses were isolated from patients with different clinical and epidemiologic patterns (4). They were isolated from patients with GE (6, 16) and neurological disorders (17–19). While GE is a threat to global health, the causative agents of many cases still remained unclear (4). Therefore, we conducted this systematic review and meta-analysis to (1) elucidate the possible role of these viruses in development of GE and (2) understand the current epidemiologic pattern of these viruses in different parts of the world.

## Methods

### Search Strategy

This systematic and meta-analysis review was performed using the recommendations of the PRISMA (Preferred Reporting Items for Systematic Reviews and Meta-Analyses) (20). We comprehensively searched from multiple electronic databases, including Web of Science, PubMed, Google scholar, and Scopus. English-language-related articles published from January 2007 to April 2021 were searched by two investigators independently (AK and MZ) using the following keywords: “Bufavirus” OR “BuV” OR “novel human picornavirus” OR “Saffold virus” OR “SAFV” OR “HCosV” OR “Human Cosavirus” AND “prevalence” OR “epidemiology” OR “molecular prevalence” AND “acute gastroenteritis” OR “diarrhea” OR “gastroenteritis” OR “gastrointestinal complications. In addition, the reference list of all relevant articles and narrative reviews were retrieved in full to search for additional eligible studies. All selected studies were imported to the EndNote software versionX8 (Thomson Reuters, California) for criteria analysis.

### Inclusion and Exclusion Criteria

The inclusion criteria for the studies were as follows: (1) All observational studies (case-control, cohort, and cross-sectional studies); (2) Published: 2007 to 2021 for SAFV, between 2012 and 2021 for BuV, and between 2008 and 2021 for HCosV; and (3) Studies reporting the molecular techniques of Bufavirus, Saffoldvirus, and Cosavirus among patients with GE across the world. Papers were excluded from this review if (1) Samples were selected entirely from patients with Bufavirus, Saffold virus, and Cosavirus; (2) Research provides incomplete data; and (3) Review articles, congress abstracts, conference papers, meta-analysis, or systematic reviews, and articles in languages other than English.

### Data Extraction

The data were extracted from 46 selected studies by two researchers separately and independently, including the first author's name, location, year of publication, continent, number of investigated patients, number of isolated viruses, target gene, molecular technique, and genotypes. If necessary, any issue related to the selection of studies was resolved by the first and corresponding authors.

### Data Synthesis and Statistical Analysis

We used a random-effect model to estimate the overall prevalence of the BuV, SAFV, and HCosV, and results are shown in the forest plot with a 95% confidence interval. Furthermore, evaluation of the prevalence of the viruses was performed on continental, country, diagnostic method, and age as well as gender subgroups. Also, the prevalence of the viruses and their association with GE were estimated and reported by odds ratio (OR). The Egger's test and *I*^2^ statistic/Cochran's Q statistic were used to determining publication bias and heterogeneity assessments, respectively, and *p* < 0.05 was considered statistically significant. All analyses of the present study were performed with comprehensive meta-analysis (V2.2, Bio stat) software.

## Results

### Search Results and Studies Characteristics

**Figure 1:**
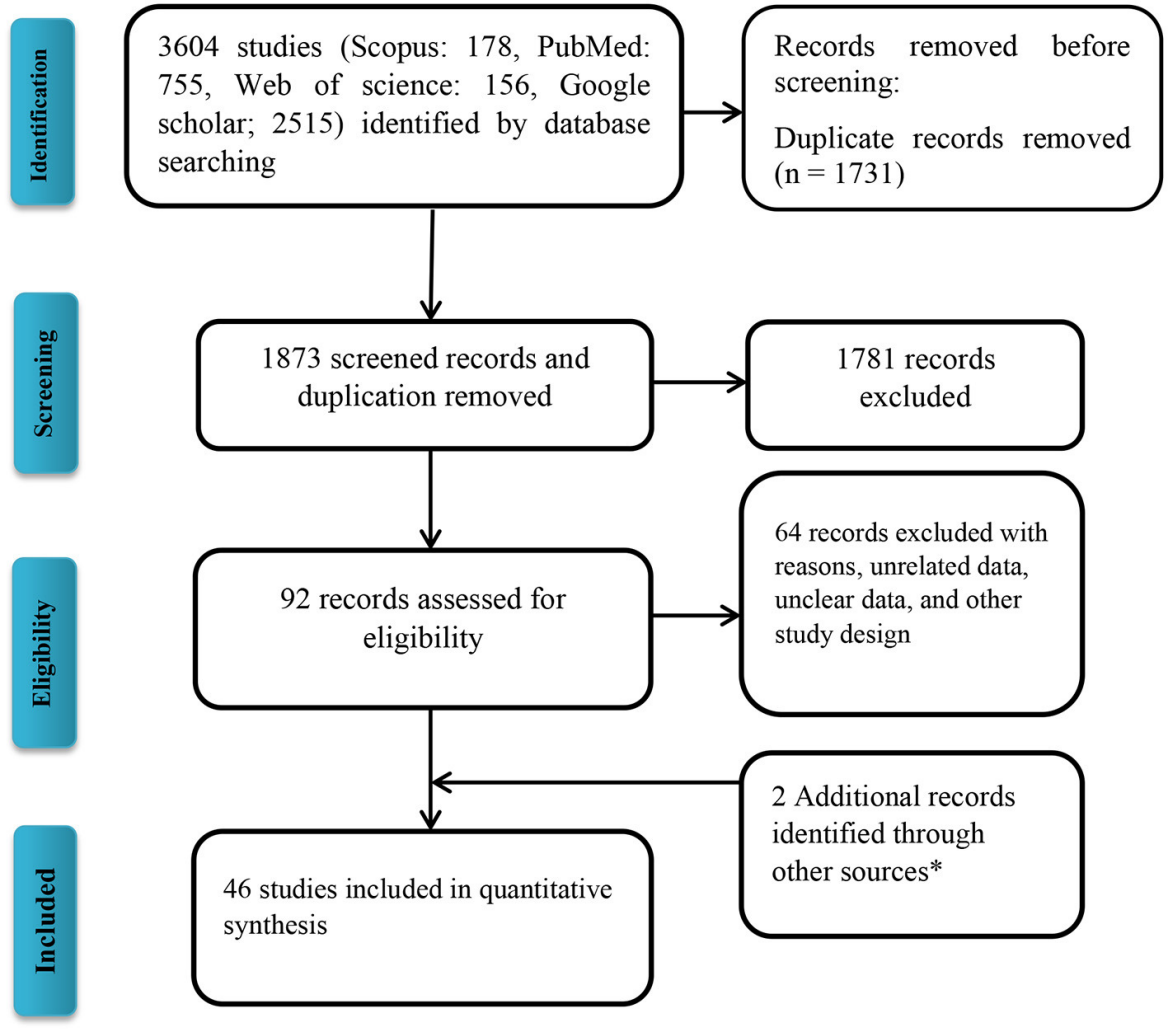
Flow diagram of the literature search for studies included in the meta-analysis. *Including manual search and library records.

Following the initial search strategy in the aforementioned databases, 3,604 original related articles were identified (PubMed: 755, Scopus: 178, Web of Science: 156, Google scholar: 2,515). A total of 46, observational articles, which included 30 cross-sectional (BuV: 6, SAFV: 12, and HCosV: 12), seven case-control (BuV: 1, SAFV: 3, and HCosV: 3), and nine cohort (BuV: 5, SAFV: 3, and HCosV: 1) studies were included based on our inclusion criteria. A summary of the research selection process and the reasons for exclusion is shown in Figure 1. In the case of Bufavirus, five articles were conducted in Europe, four in Asia, and three in Africa. About the Cosavirus, nine in Asia, four in Europe, one in Africa, and two articles were done in America. In the case of Saffold virus, 15 and three were performed in Asia Europe, respectively. Characteristics of the included 46 articles are shown in Tables 1–3.

**Table 1:** The general characterization of Bufavirus studies.

**References**	**Study type**	**Country**	**Continent**	**Publishing year**	**Cases**	**Positive**	**Target**	**Method**	**Not distinguished Genotype**	**BuV1**	**BuV2**	**BuV3**
Phan et al. (21)	Cross-sectional	Burkina Faso	Africa	2012	98	4	NS1	Nested RT-PCR		3	1	
Phan et al. (21)	Cross-sectional	Tunisia	Africa	2012	100	0	NS1	Nested RT-PCR				
Smits et al. (22)	Cross-sectional	Netherlands	Europe	2014	27	1	NS1	Real-time RT-PCR		1		
Vaisanen et al. (9)	Cross-sectional	Finland	Europe	2014	629	7	VP2	Real-time RT-PCR	7			
Yahiro et al. (23)	Cross-sectional	Bhutan	Asia	2014	393	3	NS1	Nested RT-PCR				3
Huang et al. (16)	Cross-sectional	China	Asia	2015	1877	9	NS1	Real-time RT-PCR		4		5
Altay et al. (24)	Case-control	Turkey	Europe	2015	583	8		RT-PCR				8
Chieochansin et al. (25)	Cohort	Thailand	Asia	2015	1414	1	NS1	Nested RT-PCR		1		
Chieochansin et al. (25)	Cohort	Thailand	Asia	2015	81	3	NS1	Nested RT-PCR		3		
Ayouni et al. (7)	Cohort	Tunisia	Africa	2016	203	2	NS1	Nested RT-PCR		2		
Vaisanen et al. (11)	Cohort	Finland	Europe	2016	410	3	NS1	Real-time RT-PCR	3			
Mohammad et al. (26)	Cross-sectional	Kuwait	Asia	2020	84	1		Multiplex RT-PCR				1
Dapra et al. (5)	Cohort	Italy	Europe	2021	160	0		Real-time RT-PCR				
Mohanraj et al. (27)	Cohort	Finland	Europe	2021	243	4	NS1	Multiplex real-time qPCR	4			
Mohanraj et al. (27)	Cohort	Finland	Europe	2021	386	3	NS1	Multiplex real-time qPCR	3			
Mohanraj et al. (27)	Cohort	Finland	Europe	2021	955	3	NS1	Multiplex real-time qPCR	3			
Mohanraj et al. (27)	Cohort	Latvia	Europe	2021	115	0	NS1	Multiplex real-time qPCR	0			
Mohanraj et al. (27)	Cohort	Malawi	Africa	2021	164	1	NS1	Multiplex real-time qPCR	1			

**Table 2:** The general characterization of Saffold virus studies.

**References**	**Study**	**Country**	**Continent**	**Publishing year**	**Cases**	**Positive**	**Target**	**Method**	**SAFV-1**	**SAFV-2**	**SAFV-3**	**SAFV-4**	**SAFV-6**
Ren et al. (28)	Cross-sectional	China	Asia	2009	373	12	5′ UTR	Nested RT-PCR	12				
Khamrin et al. (29)	Cross-sectional	Thailand	Asia	2011	150	4	5′ UTR	Nested RT-PCR		4			
Dai et al. (30)	Case-control	China	Asia	2011	577	6	5′ UTR	Nested RT-PCR			3		
Zhang et al. (31)	Cohort	China	Asia	2012	2,013	12	5′ UTR	Real-time RT-PCR		4	5		
Khamrin et al. (32)	Cross-sectional	Japan	Asia	2013	454	7	5′ UTR	Nested RT-PCR		5	2		
Nielsen et al. (33)	Cohort	Denmark	Europe	2013	386	10	VP1	Real-time RT-PCR		10			
Yodmeeklin et al. (34)	Cross-sectional	Thailand	Asia	2015	608	9	5′ UTR	Nested RT-PCR	1	5	2	1	
Thongprachum et al. (35)	Cross-sectional	Japan	Asia	2017	751	4	5′ UTR	Multiplex RT-PCR					
Kumthip et al. (36)	Cross-sectional	Thailand	Asia	2017	73	1	5′ UTR	Nested RT-PCR					
Menage et al. (6)	Cross-sectional	Thailand	Asia	2017	1,093	18	5′ UTR	Nested RT-PCR	3	9			6
Li et al. (37)	Case-control	China	Asia	2017	461	7	VP1	Nested RT-PCR	3	4			
Dapra et al. (38)	Cross-sectional	Italy	Europe	2018	164	1		NR^*^					
Malasao et al. (39)	Cross-sectional	Thailand	Asia	2019	2,002	30		NR					
Kim et al. (40)	Cross-sectional	South Korea	Asia	2020	801	0		Multiplex RT-PCR					
Mohammad et al. (26)	Cross-sectional	Kuwait	Asia	2020	84	1		Metagenomics sequencing					
Vandesande et al. (41)	Cohort	Sweden	Europe	2021	209	11	5′ UTR	Semi-nested RT-PCR			1		
Yaghobi et al. (42)	Cross-sectional	Iran	Asia	2020	160	26	5′ UTR	RT-PCR					
Taghinejad et al. (43)	Cross-sectional	Iran	Asia	2020	160	11		RT-PCR					

* *NR, Not reported*.

**Table 3:** The general characterization of Cosavirus studies.

**References**	**Study**	**Publishing year**	**Country**	**Continent**	**Cases**	**Positive**
Nielsen et al. (33)	Cohort	2013	Denmark	Europe	386	0
Stocker et al. (44)	Case-control	2012	Brazil	America	359	13
Vizzi et al. (45)	Case-control	2021	Venezuela	America	82	5
Yu et al. (46)	Case-control	2017	China	Asia	461	8
Ayouni et al. (7)	Cross-sectional	2016	Tunisia	Africa	203	2
Dapra et al. (38)	Cross-sectional	2018	Italy	Europe	164	0
Dapra et al. (5)	Cross-sectional	2021	Italy	Europe	160	0
Khamrin et al. (47)	Cross-sectional	2012	Thailand	Asia	300	1
Khamrin et al. (48)	Cross-sectional	2014	Thailand	Asia	411	1
Kim et al. (40)	Cross-sectional	2020	South Korea	Asia	801	0
Menage et al. (6)	Cross-sectional	2017	Thailand	Asia	1,093	16
Mohammad et al. (26)	Cross-sectional	2020	Kuwait	Asia	84	1
Okitsu et al. (49)	Cross-sectional	2014	Japan	Asia	630	1
Rovida et al. (50)	Cross-sectional	2013	Italy	Europe	689	1
Thongprachum et al. (35)	Cross-sectional	2017	Japan	Asia	751	1
Kochjan et al. (51)	Cross-sectional	2016	Thailand	Asia	21	1

### Pooled Prevalence of Bufavirus in the Patients With Gastroenteritis

**Figure 2:**
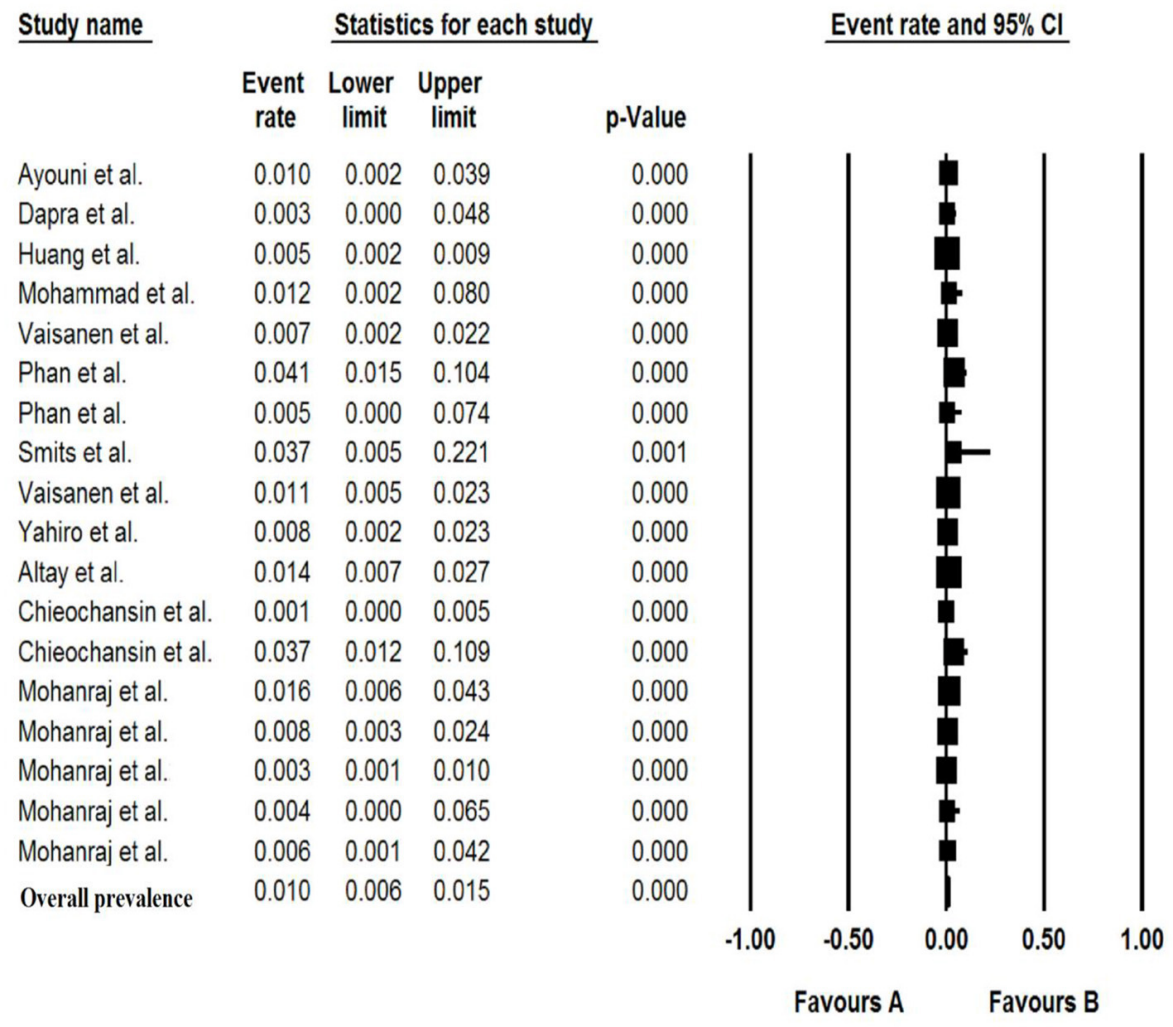
Forest plot of the pooled prevalence for BuV.

The total number of patients with GE included in this meta-analysis was 7,922 from children and adults based on 11 articles. The pooled prevalence of Bufavirus infection among patients with GE was 1.% (95% CI, 0.6–1.5%) based on a random-effects meta-analysis (Figure 2). In subgroup analysis by continent, the highest prevalence of Bufavirus was seen in Africa (1.4%, 95% CI, 0.5–4.1%) while the lowest prevalence was observed in Asia (0.7%, 95% CI, 0.2–2.1%) (Table 4). Highest prevalence of virus belongs to older than 5 years old subgroups (3.7%, 95% CI: 1.4–9.5%). As well, in three genotypes of BuV, BuV1 (1.%, 95% CI: 0.3–3.4%), and BuV2 (1.%, 95% CI: 0.1–6.9%) were of the same prevalence, while BuV3 (0.7%, 95% CI: 0.3–1.7%) was less prevalent.

**Table 4:** The Bufavirus prevalence based on subgroups and studies heterogeneity.

**Characteristics**	**Categories**	**Data sets**	**Pooled prevalence (%)** **(95% CI)**	**Heterogeneity**		
				***Q* value**	***P*-value**	***I*^2^%**
Overall	–	18	1.0 (0.6–1.5)	35.005	0.006	51.435
Continent	Africa	4	1.4 (0.5–4.1)	5.486	0.139	45.319
	Asia	5	0.7 (0.2–2.1)	15.201	0.004	73.685
	Europe	9	1.0 (0.7–1.4)	9.203	0.325	13.071
Method	Nested RT-PCR	5	1.1 (0.4–3.1)	18.311	0.003	72.694
	Real-time RT-PCR	5	0.8 (0.4–1.4)	5.853	0.210	31.660
	multiplex real-time qPCR	5	0.7 (0.4–1.4)	4.975	0.290	19.599
Genotype	BuV1	6	1.0 (0.3–3.4)	27.351	0.000	81.719
	BuV2	1	1.0 (0.1–6.9)	0.000	1.000	0.000
	BuV3	4	0.7 (0.3–1.7)	8.548	0.036	0.501
Co–infection	NoV	6	0.3 (0.1–0.5)	4.103	0.535	0.000
	HBoV	2	0.3 (0.1–0.9)	0.078	0.780	0.000
	RoV	2	0.6 (0.2–2.2)	1.307	0.253	23.480
	AdV	1	1.0 (0.2–3.9)	0.000	1000	0.000
Age	Under 5	5	1.4 (0.6–2.9)	7.381	0.117	45.804
	Over 5	2	3.7 (1.4–9.5)	0.000	1.000	0.000
Sex	Male	4	0.9 (0.2–4.4)	12.447	0.006	75.898
	Female	4	0.6 (0.2–1.8)	4.279	0.233	29.883

### The Association of Bufavirus With Gastroenteritis

**Figure 3:**
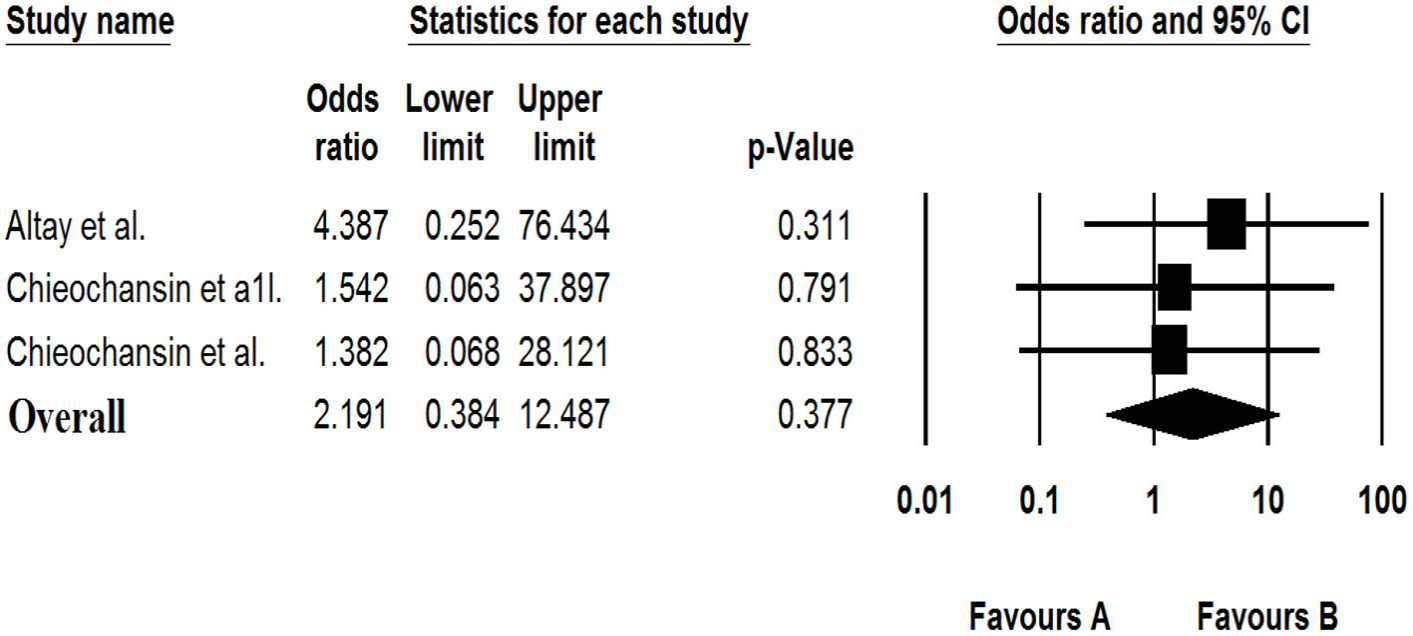
Forest plot of odds ratios for the BuV based on case-control studies.

In three data sets, the meta-analysis showed that Bufavirus was not associated with GE [OR: 2.191 (95% CI; 0.384–12.487), *I*^2^: 0%] (Figure 3).

### Pooled Prevalence of Saffold Virus in the Patients With Gastroenteritis

**Figure 4:**
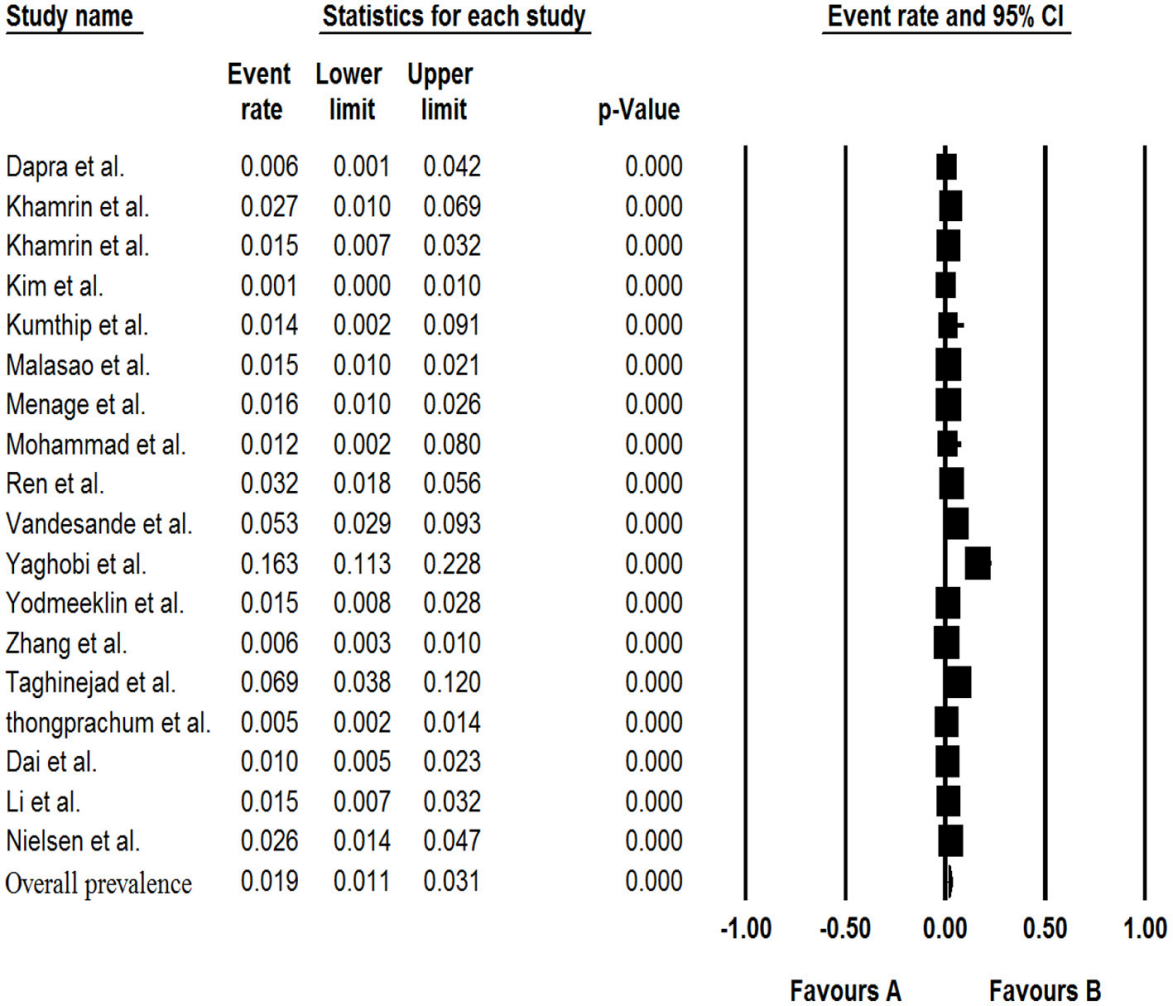
Forest plot of the pooled prevalence for SAFV.

The results of analysis of Saffold virus based on random-effects meta-analysis are summarized in Table 4. Using random-effects meta-analysis, the pooled prevalence of Saffold virus in the studied patients was 1.9% (95% CI, 1.1–3.1%) (Figure 4). Among included studies, the maximum and minimum pooled prevalence of Saffold virus among patients with GE was found in Europe and Asia, respectively (2.9, 95% CI: 1.2–6.5% vs. 1.7, 95% CI: 0.9–3.1%) (Table 5). The highest prevalence of the virus was detected in children younger than 5 years of old (2.4%, 95% CI: 0.6–0.9). Among the eight genotypes of SAFV, SAFV-2 was the most prevalent genotype (1.%, 95% CI: 0.5–1.9%), and SAFV-4 was the least prevalent (0.2%, 95% CI: 0–1.2%) in patients with GE.

**Table 5:** The Saffold virus prevalence based on subgroups and studies heterogeneity.

**Characteristics**	**Categories**	**No. of** **Datasets**	**Pooled prevalence (%)** **(95% CI)**	**Heterogeneity**		
				***Q* value**	***P*-value**	***I*^2^%**
Overall	–	18	1.9 (1.1–3.1)	174.465	0.000	90.256
Continent	Asia	15	1.7 (0.9–3.1)	165.693	0.000	91.553
	Europe	3	2.9 (1.2–6.5)	5.965	0.051	66.471
Genotype	SAFV-1	5	0.9 (0.3–2.6)	25.159	0.000	84.101
	SAFV-2	7	1.0 (0.5–1.9)	23.800	0.001	74.790
	SAFV-3	6	0.6 (0.2–1.5)	23.853	0.000	79.038
	SAFV-4	1	0.2 (0.0–1.2)	0.000	1.000	0.000
	SAFV-6	1	0.5 (0.2–1.2)	0.000	1.000	0.000
Co-infection	NoV	6	0.6 (0.3–1.0)	8.635	0.125	42.097
	HBoV	2	0.4 (0.1–1.5)	1.457	0.227	31.352
	RoV	8	0.4 (0.2–0.9)	19.395	0.007	63.909
	AdV	4	0.2 (0.1–0.5)	2.624	0.453	0.000
Method	Multiplex RT-PCR	2	0.3 (0.0–1.9)	2.052	0.152	51.263
	Nested RT-PCR	7	2.3 (1.5–3.5)	14.417	0.025	58.383
	RT-PCR	2	10.9 (4.6–24.)	6.505	0.011	84.627
Age	Under 5	8	1.6 (0.5–4.5)	70.138	0.000	90.020
	Over 5	3	2.4 (0.6–0.9)	4.183	0.124	52.184
Sex	Male	2	0.3 (0.0–2.2)	0.984	0.321	0.000
	Female	2	0.9 (0.0–19.7)	3.846	0.050	73.999

### The Association of Saffold Virus With Gastroenteritis

**Figure 5:**
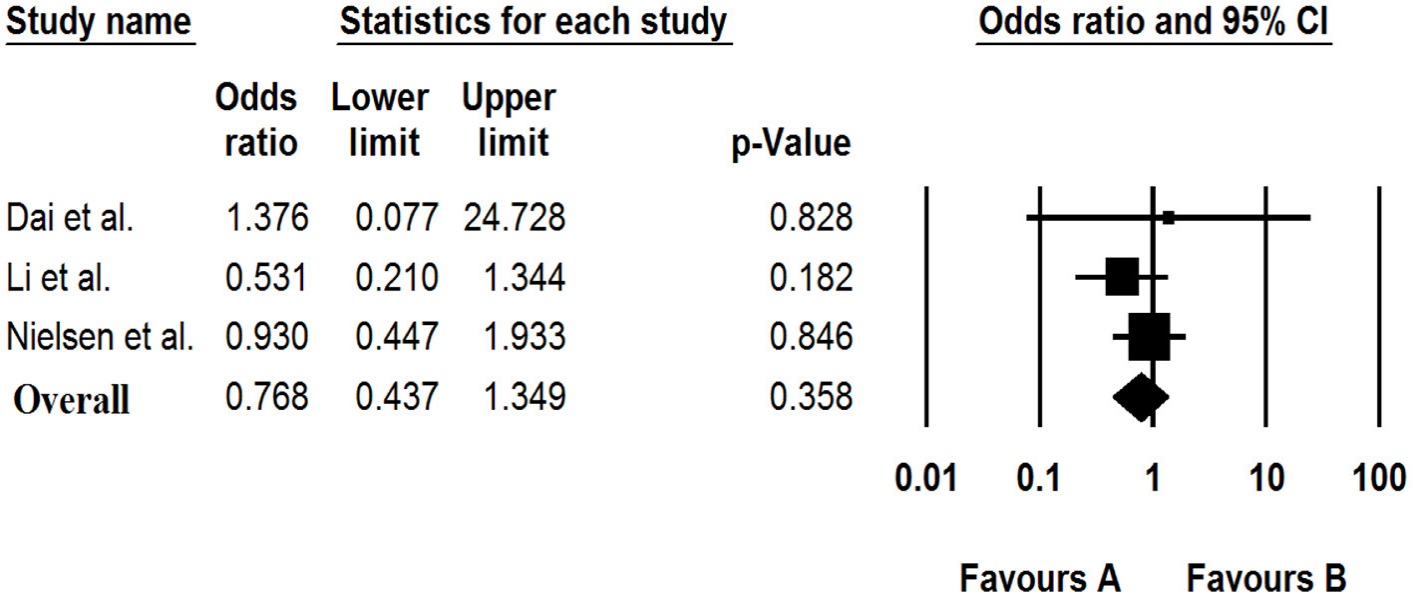
Forest plot of odds ratios for the SAFV based on case-control studies.

Based on the meta-analysis of three case-control studies, there was no significant association between the Saffold virus and GE [OR: 0.768 (95% CI: 0.437–1.349), *I*^2^: 0%] (Figure 5).

### Pooled Prevalence of Human Cosavirus in the Patients With Gastroenteritis

**Figure 6:**
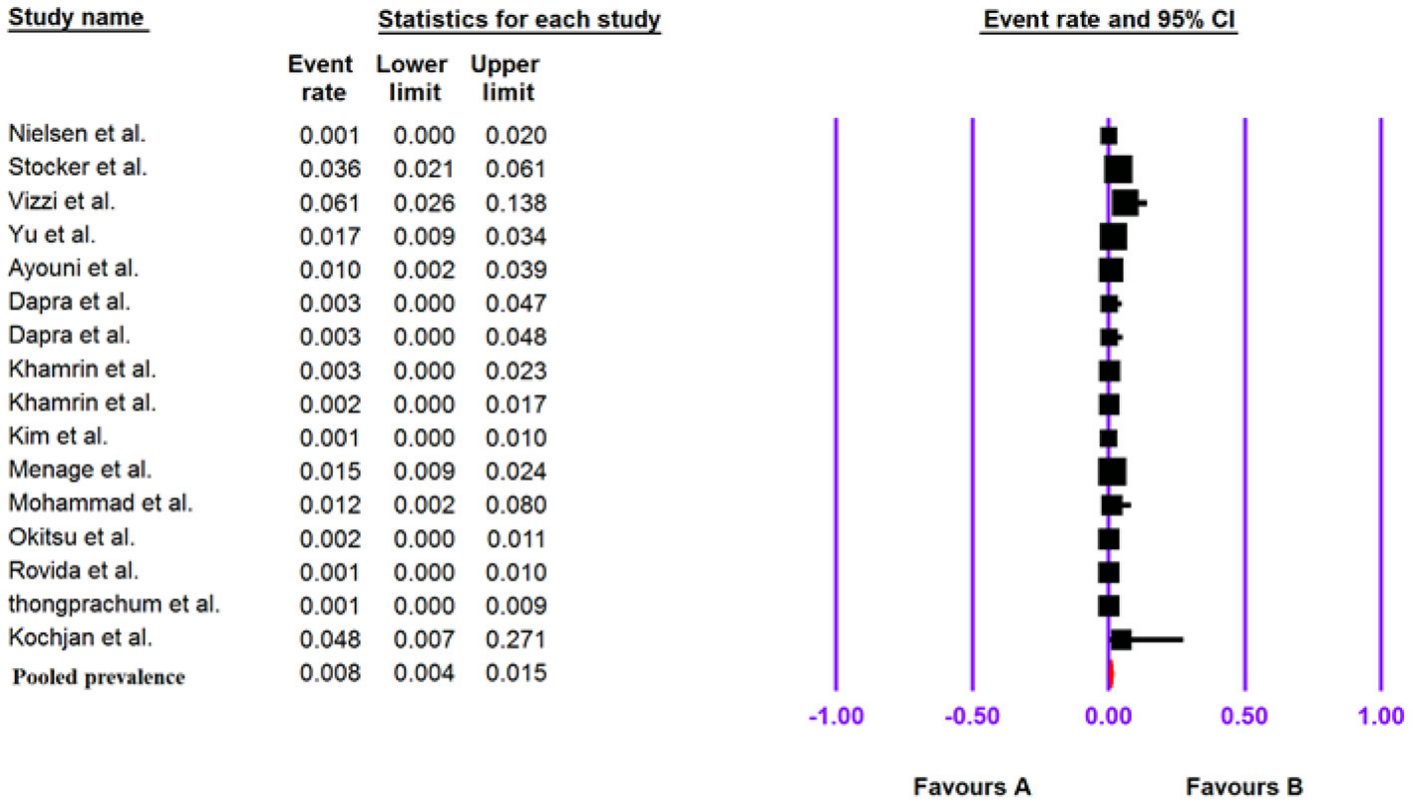
Forest plot of the pooled prevalence for HCosV.

The total number of patients with GE included in this meta-analysis was 6,595 based on 16 included articles. Based on a random-effect meta-analysis, the pooled prevalence of the human Cosavirus infection among patients with GEs was 0.8% (95% CI, 0.4–1.5%) (Figure 6). In subgroup analysis by continent, the highest prevalence of Cosavirus was seen in America (4.2%, 95% CI, 2.6–6.6%), whereas Europe (0.2%, 95% CI, 0.1–0.7%) observed the lowest prevalence (Table 6).

**Table 6:** The Cosavirus prevalence based on subgroups and studies heterogeneity.

**Characteristics**	**Categories**	**No. of** **Data sets**	**Pooled prevalence (%)** **(95% CI)**	**Heterogeneity**		
				***Q* value**	***P*-value**	***I*^2^%**
Overall	–	16	0.8 (0.4–1.5)	28.29	0.000	92.932
WHO regions	Africa	1	1.0 (0.2–3.9)	0.000	1.000	0.000
	America	2	4.2 (2.6–6.6)	1.022	0.312	2.185
	Asia	9	0.7 (0.3–1.4)	21.240	0.007	62.335
	Europe	4	0.2 (0.1–0.7)	0.377	0.945	0.000
Genotype	HCoSV-A	3	0.5 (0.1–2.1)	6.292	0.043	68.213
	HCoSV-C	1	0.1 (0.0–0.6)	0.000	1.000	0.000
	HCoSV-D	2	0.2 (0.0–0.7)	0.837	0.360	0.000
Co-infection	NoV	2	0.2 (0.0–1.1)	1.420	0.233	29.561
	EV	3	0.7 (0.1–3.3)	5.932	0.052	66.286
	RoV	3	0.4 (0.2–0.8)	1.384	0.500	0.000
	AdV	5	0.6 (0.1–2.1)	9.329	0.053	57.122
Age	<5	10	0.5 (0.2–1.1)	21.031	0.013	57.207
	<15	7	1.2 (0.5–2.9)	18.564	0.005	67.680
	>15	2	0.4 (0.1–1.8)	0.319	0.517	0.000

### The Association of Human Cosavirus With Gastroenteritis

**Figure 7:**
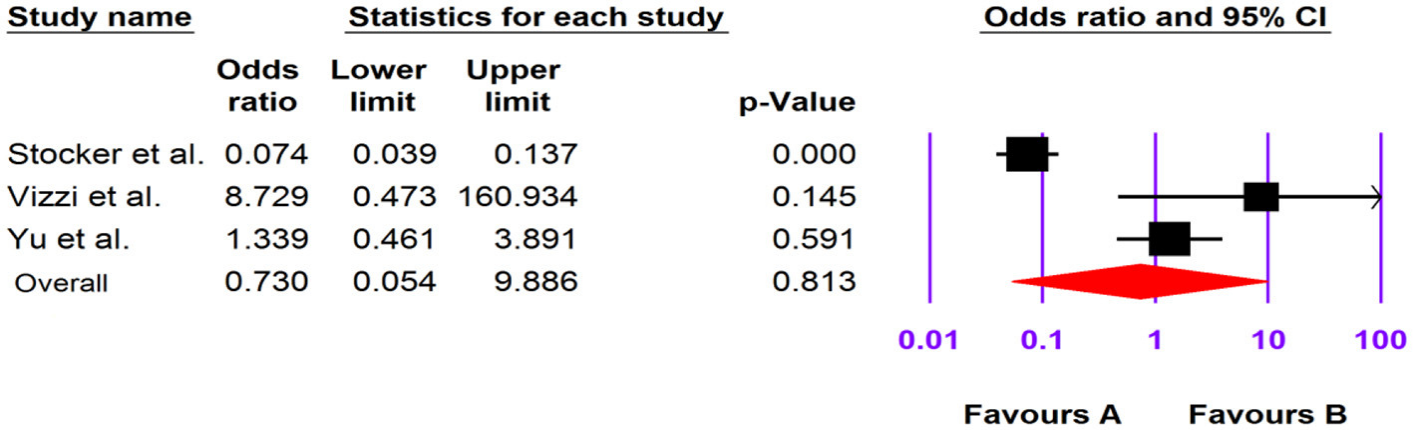
Forest plot of odds ratios for the HCosV based on case-control studies.

Of the four included case-control studies, one study could not be analyzed due to zero values for cases and controls (33), and, according to the three analyzed studies, human Cosavirus was not associated with GE [OR: 0.730 (95% CI; 0.054–9.886), *I*^2^: 0%] (Figures 3, 7).

### Publication Bias and Heterogeneity Assessment

**Figure 8:**
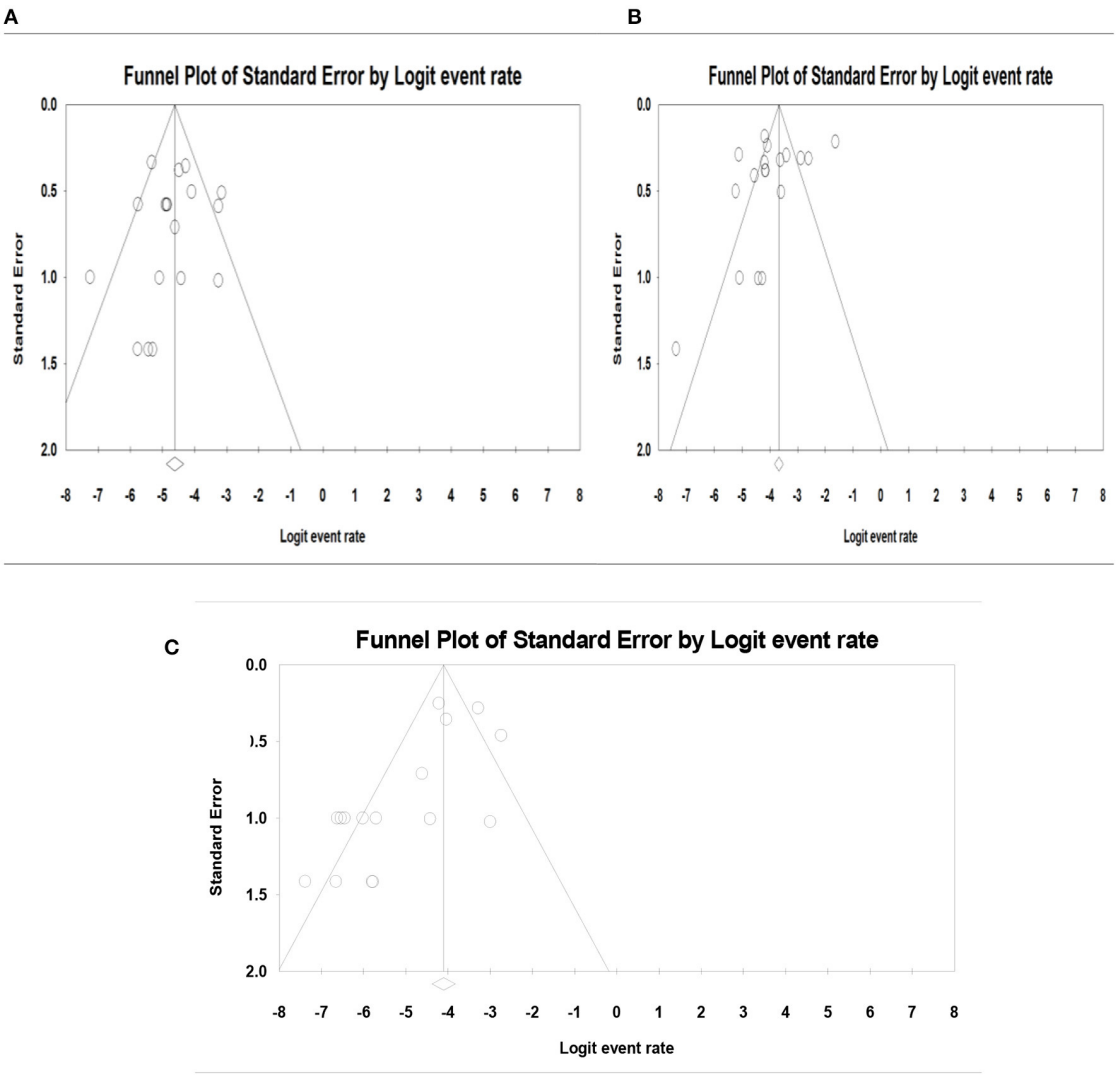
Funnel plot for publication bias assessment in BuV **(A)**, SAFV **(B)**, and CosV **(C)**.

The publication bias results were not significant for two viruses (SAFV and BuV) and significant for Cosavirus prevalence reports by applying Egger's regression test (*P* = 0.1912 for SAFV, *P* = 0.5667 for BuV, vs. *P* = 0.0031 for Cosavirus) (as shown in Figure 8). Also, the heterogeneity results of the studies according to the *I*^2^ statistics and Cochran's Q statistics were statistically significant for BuV (*Q* = 35.005, *p* < 0.006, *I*^2^ = 51.435%), SAFV (*Q* = 174.465, *p* < 0, *I*^2^ = 90.256%), and Cosavirus (*Q* = 28.29, P = 0, *I*^2^ = 92.932) (Tables 4–6).

## Discussion

Rapid progressions in sequencing technologies, bioinformatics, and metagenomic have led to the discovery of new viruses in recent years. However, while some studies stated the isolation of new viruses from fecal samples of patients with GE, there is still no solid evidence of the association of these viruses with GE (4, 52, 53). They are often neglected in epidemiological studies as they cause milder or asymptomatic infection, and researchers have a higher tendency to detect common enteric viruses and other infectious agents in patients with GE (54–56). In the present meta-analysis, we investigated the role of three emerging discovered viruses in the development of GE. Our results show no association between infection with Bufavirus (OR; 2.91, 95% CI: 0.384–12.487), Cosavirus (OR; 0.73, 95% CI: 0.054–9.886), and Saffold virus (OR; 0.77, 95% CI: 0.44–1.35) with GE. Also, a low prevalence of BuV (1.%, 95% CI: 0.6–1.5%), HCoSV (0.8%, 95% CI: 0.4–1.5%), and SAFV (1.9%, 95% CI: 1.1–3.1%) was observed. In general, the prevalence of SAFV was higher than BuV, and the least prevalence was observed in the case of HCoSV. The highest prevalence of BuV was in Africa (1.4%, 95% CI: 0.5–4.1%), where it was discovered (21), and the least prevalence was in Asia (0.7%, 95% CI: 0.2–2.1%). This might be due to poor hygiene and lack of access to safe water in African countries. Given the fact that these viruses were detected in environmental and sewage samples from various parts of the world (57–62), they possibly transmit through the oral-fecal route.

About the three genotypes of BuV, BuV1, and BuV2 were of the same prevalence, while BuV3 was less common in patients with GE; this lower prevalence of BuV3 might be due to the later discovery of this genotype in 2014 (23). SAFV consists of eight genotypes, of which five (SAFV1-4 and 6) were found in the included studies. SAFV-2 was the most prevalent genotype, and SAFV-4 was the least prevalent in patients with GE. It should be pointed out that, although SAFV genotypes 5, 7, and 8 were not detected in the included studies, Blinkova et al. isolated them along with other genotypes in children with non-polio acute flaccid paralysis (63). Also, some of the included studies did not investigate the genotypes of isolated SAFVs. Therefore, we cannot conclude that they are not present in fecal samples of patients with GE. The genotype A of HCoSV was more frequently (0.5%, 95% CI: 0.1–2.1%) isolated from patients with GE. Other founded genotypes were Genotype D (0.2%, 95% CI: 0–0.7%) and C (0.1%, 95% CI: 0–0.6%).

The presence of common enteric viruses, such as Rotavirus (RoV), human bocavirus (HBoV), Adenovirus (AdV), and Norovirus (NoV), was observed in patients that are BuV and SAFV infected. According to the Tables 4–6, co-infection with NoV was more common in patients infected with SAFV than BuV. There was a similar situation in the case of HBoV in which more prevalence of this virus was seen in SAFV than patients who are BuV infected. Contrastingly, RoV infection was more frequent in patients infected with BuV than SAFV. Similarly, AdV infection was more common in patients with BuV than SAFV infection. EVs have the highest proportion of co-infection with HCoSV followed by AdVs, RoVs, and NoVs. The high rate of co-infection with classic enteric viruses may indicate the role of these viruses in causing symptoms in patients infected with these newly discovered viruses (6, 46). The other possible point that is against the pathologic role of these viruses in the development of GE is the low viral load in patients with GE, which might be due to transient infection and the lack of replication in the gastrointestinal tract (44). Also, the high presence of these viruses in healthy individuals raises the likelihood that they are a part of the human virome (6).

Three studied viruses can infect people of all age groups (16, 41). Our analysis showed that BuV and SAFV are more common in individuals older than 5 years of age. In contrast, HCoSV was more common in the children younger than 15 years old. While GE is known as a prevalent disease in children younger than 5 years of age and common enteric viruses such as RoV and NoV are mostly found in this age group (64, 65), interestingly, our analysis showed that these viruses are more prevalent in older patients. These results might be due to reason that outdoor activities further expose people to viral agents (52).

BuV and SAFV are differently distributed among males and females, while BuV is more prevalent in males than females; SAFV is more common in females (42). However, these slight differences do not implicate that these viruses have a higher tendency to infect people of a specific gender.

All included studies had a molecularly based diagnosis with relatively close sensitivity and specificity. However, in the case of SAFV, RT-PCR had the highest detection, while nested-PCR showed the highest detection rate for BuV. It is noteworthy to mention that it requires more studies on the sensitivity and specificity of these methods to conclude which one is more suitable.

The present study faced some limitations. There were a few studies on adults, and details of participants (gender, clinical signs, and age groups) were insufficient in some studies. The genotypes of the viruses were not reported from some studies, and also some of research conducted without a healthy control group. The prevalence of these viruses had not been reported in many countries and geographical areas. In addition, some of the included studies did not evaluate the co-infection of the novel viruses with common enteric viruses. In addition, the language limitations of many studies and lack of association assessments of genotypes and clinical signs were the other main limitations of the present study. Hence, we suggest further studies, especially in case-control design, and more comprehensive studies from different geographical areas to overcome these limitations.

## Conclusion

Progression in the development of molecular and metagenomics methods has facilitated discovering and studying emerging viruses. In the present meta-analysis, we investigated the prevalence and role of three recently discovered viruses in the development of GE. The pooled prevalence of three viruses was low, and neither was associated with GE. These results might be due to the few numbers of studies conducted. Therefore, we suggest more comprehensive studies with large cohorts of symptomatic and healthy patients in order to enhance our knowledge about these newly identified viruses. Also, we recommend *in vitro* studies to investigate the possible effects of these viruses on the gastrointestinal cell lines. In addition, the possible role of these emerging viruses in the etiology of other complications, such as respiratory symptoms, neurological diseases, and fever of an unknown origin, should not be neglected.

## Data Availability Statement

The original contributions presented in the study are included in the article/supplementary materials, further inquiries can be directed to the corresponding author/s.

## Author Contributions

MR and AK designed the study and collaborated in the manuscript writing. MR and MZ collaborated in the studies search, data extraction, and double checking. MZ helped in revision. All authors commented on the drafts of the manuscript and approved the final version of the article.

## Conflict of Interest

The authors declare that the research was conducted in the absence of any commercial or financial relationships that could be construed as a potential conflict of interest.

## Publisher's Note

All claims expressed in this article are solely those of the authors and do not necessarily represent those of their affiliated organizations, or those of the publisher, the editors and the reviewers. Any product that may be evaluated in this article, or claim that may be made by its manufacturer, is not guaranteed or endorsed by the publisher.
